# Are Neutrophil-to-Lymphocyte and Platelet-to-Lymphocyte Ratios Associated with Mortality in Pediatric Trauma Patients? A Retrospective Study

**DOI:** 10.5041/RMMJ.10376

**Published:** 2019-10-29

**Authors:** Yusuf Kenan Tekin

**Affiliations:** Department of Emergency, Sivas Cumhuriyet University, İmaret/Merkez/Sivas, Turkey

**Keywords:** Neutrophil-to-lymphocyte ratio, pediatric patient, platelet-to-lymphocyte ratio, trauma

## Abstract

**Background:**

There are very limited data on the prognostic capacity of the neutrophil-to-lymphocyte ratio (NLR) and platelet-to-lymphocyte ratio (PLR) for the systemic inflammatory response in pediatric trauma (PT) patients. The purpose of this study was to evaluate the prognostic ability of NLR and PLR on mortality in pediatric trauma patients.

**Methods:**

This study looked at 358 PT patients who were admitted to the Cumhuriyet University Hospital’s Emergency Department between January 2010 and June 2018. The NLR and PLR were calculated by dividing the blood neutrophil count and blood platelet count, respectively, by the lymphocyte count, at the time of admission. After performing a stepwise logistic regression analysis to determine the predictive factors on the mortality risk of post-traumatic systemic inflammatory response syndrome (SIRS), receiver operating characteristic (ROC) curve analysis was used to define the optimum cut-off values of the NLR and the PLR parameters for survival.

**Results:**

The NLR, and PLR values were significantly higher in survivors than in non-survivors (NLR, 6.2±5.7 versus 2.6±2.5, *P*<0.001; PLR, 145.3±85.0 versus 46.2±25.2, P<0.001 ). The NLR (odds ratio [OR], 3.21; *P*=0.048), PLR (OR, 0.90; *P*=0.032), blood glucose (OR, 1.02; *P*=0.024), and Injury Severity Score (ISS) (OR, 1.28; *P*=0.011) were independent predictors of the mortality risk in PT patients. The area under the curve in the ROC curve analysis was 0.764 with a cut-off of 2.77 (sensitivity 70%, specificity 77%) for the NLR; and 0.928 with a cut-off of 61.83 (sensitivity 90%, specificity 85%) for the PLR.

**Conclusion:**

Acquiring the NLR and PLR at the time of admission could be a useful predictor for mortality in PT patients.

## INTRODUCTION

Trauma-related injury as a potential cause of death affects millions of people worldwide, especially in less developed countries.^1^ Moreover, it is the leading cause of mortality in pediatric trauma patients.^2^,^3^ Severe trauma-related injuries may also promote a hyper-inflammatory state (usually characterized by the presence of systemic inflammatory response syndrome [SIRS]) leading to multi-organ dysfunction syndrome (MODS), which is associated with increased mortality.^4^,^5^

Systemic inflammatory response syndrome is usually found in multi-trauma patients on at least one day during the first 72 h.^5^ Although it is clear that SIRS is frequently observed after severe trauma and is related to adverse health outcomes in the adult population, the effects of early SIRS and changes in the endothelial physiology in pediatric trauma (PT) patients are not fully understood.^4^ There are restricted data on the proposed mechanisms of trauma-induced SIRS in the pediatric population. Unfortunately, the current understanding of the possible mechanisms in SIRS is largely based on data derived from the adult population. However, in addition to the physiological and anatomical differences between children and adults, there is a variance in patterns and mechanisms of injury.^6^

In previous research, several scoring systems and a variety of biomarkers, such as Boehme’s score system and IL-6 and the histone-complexed DNA (hcDNA), were explored to stratify mortality risk in trauma patients.^7^,^8^ These paradigms proved beneficial for medical triage, further resuscitation of patients, and better patient management. But only a few of these scoring systems have been used in practice routinely, partly because of relatively low clinical practicality.

The most common tests, white blood cell (WBC) count and whole blood assay, have long been used to assess SIRS in clinical practice.^9^ To this day, the neutrophil-to-lymphocyte ratio (NLR) and the platelet-to-lymphocyte ratio (PLR) (as a new marker of inflammatory status) have been investigated to examine the prediction ability of systemic inflammatory response in many other disorders, including malignant cancers and pulmonary diseases.^10^–^12^ Nevertheless, very limited data have been presented on the prognostic capacity of both NLR and PLR for SIRS in PT patients.

## MATERIAL AND METHODS

This retrospective study included a total of 358 PT patients admitted to the emergency department (ED) of Cumhuriyet University Hospital, between January 2010 and June 2018, due to acute trauma.

The inclusion criteria were: patients (1) with acute trauma, and (2) under the age of 19 years at the time of ED admission. Exclusion criteria were as follows: (1) patients aged 19 years and older; (2) inaccessible or missing data from the hospital system; (3) patients admitted more than 24 hours after the occurrence of trauma; (4) patients presenting with any of the following co-morbid or chronic conditions: liver disease, renal failure, uncured malignant disease of any origin, immunodeficiency, pregnancy, coagulation disorders, and/or diagnosis of SIRS or infectious disease at the time of admission.

The present study was reviewed and approved by the Ethics Committee of Cumhuriyet University (protocol number 2018-11/04) and conducted in accordance with the principles of the Declaration of Helsinki. Informed consent was received from the patient and/or the patient’s legal guardian.

Demographic and medical data such as the Injury Severity Score (ISS) or injury type were calculated or obtained at the time of admission.

Data for hemoglobin (Hb), neutrophil, monocyte, white blood cell (WBC), lymphocyte, blood glucose, alanine aminotransferase (ALT), aspartate aminotransferase (AST), activated partial thromboplastin time (aPTT), and international normalized ratio (INR) were retrospectively derived from the medical record database of the first electronic application of biochemical results.

Both the NLR and PLR were calculated at the time of admission by dividing the blood neutrophil count and the blood platelet count, respectively, by the lymphocyte count. The data regarding absolute neutrophil count and absolute lymphocyte count were obtained from the first routine blood assay at the time of admission.

### Statistical Analysis

Data were analyzed with the SPSS software version 22.0 for Windows (IBM Corporation, New York, NY, USA). Continuous variables were presented as mean±standard deviation (SD) as appropriate, and categorical variables as numbers (percentages). The Mann–Whitney *U* test was used for continuous variables, and categorical variables were compared with the Fisher exact test, to assess the significance of intergroup differences.

After performing a stepwise logistic regression analysis to determine the predictive factors for the mortality risk of post-traumatic SIRS, a receiver operating characteristic (ROC) curve analysis and associated areas under the curve (AUCs) with 95% CIs were separately plotted to define the optimum cut-off point both for the NLR and the PLR. A *P* value <0.05 was considered statistically significant.

## RESULTS

A total of 358 PT patients (Table 1) were recruited in this study. Nine patients (2.5%) died within 24 hours without undergoing surgical intervention. Seventy-eight (21.8%) patients underwent surgery, with the most frequent operations being to the extremities, cranium, chest, and/or abdomen (data not shown). Four of the patients (4/78, 5.1%) died between 24 hours and 7 days following surgery.

**Table 1:** Demographic Characteristics of the Survivors, Non-survivors, and Total Sample.

Characteristics	Total Sample (*n*=358)	Survivors (*n*=345)	Non-survivors (*n*=13)	*P* Value (Fisher’s exact test)
Age, year (mean±SD)	8.96±5.51	8.98±5.52	8.62±5.38	0.798*
Gender, *n* (%)				
Male	235 (65.6)	226 (34.5)	4 (30.8)	0.100
Female	123 (34.4)	119 (65.5)	9 (69.2)	
Mechanisms of injuries, *n* (%)				
Traffic accident	293 (81.8)	280 (81.2)	13 (100.0)	0.137
Others†	65 (28.2)	65 (18.8)	0 (00.0)	

* Mann-Whitney *U* test.

† Including falling and penetrating injuries.

Table 1 shows that there was no significant difference between the two groups of survivors and non-survivors in terms of gender, age, and traffic accident as injury mechanism (male, 226/4, female, 119/9; mean age [SD], 8.98 [5.52] versus 8.62 [5.38]; and 280/13, respectively, *P*>0.05).

Referring to Table 2, the Hb, monocyte, NLR, and PLR levels were found to be statistically higher in survivors than in non-survivors (Hb, mean [SD] 12.9 [1.7] g/dL versus 10.7 [2.3] g/dL, *P*<0.001; monocyte, 1.2 [6.5] 10^3^/μL versus 1.0 [0.9] 10^3^/μL, *P*<0.001; NLR, 6.2 [5.7] versus 2.6 [2.5], *P*<0.001; PLR, 145.3 [85] versus 46.2[25.2] *P*<0.001, respectively). Furthermore, mean values of the WBC, neutrophil, lymphocyte, blood glucose, ALT, AST, aPTT, INR levels and ISS scores were higher in non-survivors than in survivors (WBC, mean [SD] 15.3 [5.7] 10^3^/μL versus 25.0 [13.7] 10^3^/μL, *P*=0.026; neutrophil, 11.8 [5.3] 10^3^/μL versus 15.0 [6.9] 10^3^/μL, *P*=0.007; lymphocyte, 3.0 [2.0] 10^3^/μL versus 9.3 [8.7] 10^3^/μL, *P*=0.001; blood glucose, 124.7 [37.3] mg/dL versus 289.9 [145.6] mg/dL, *P*=0.002; ALT, 54.5 [110.2] IU/L versus 273.2 [309.7] IU/L, *P*=0.026; AST, 100.0 [196.5] IU/L versus 507.3 [577.6] IU/L, *P*=0.026; aPTT, 28.8 [4.4] s versus 37.9 [12.4] s, *P*=0.020; INR, 1.1 [0.1] versus 1.5 [0.4], *P*=0.004; and ISS, 11.7 [7.4] versus 37.0 [12.7], *P*<0.001, respectively). However, there was no significant difference between survivors and non-survivors in terms of lymphocyte-to-monocyte ratio and mean platelet volume-to-platelet count values.

**Table 2:** Mean Values of Blood Work for Survivors versus Non-survivors.

Variables	Survivors (*n*=345, mean±SD)	Non-survivors (*n*=13, mean±SD)	*P* value (Mann-Whitney *U* test)
White blood cell (10^3^/μL)	15.3±5.7	25.0±13.7	0.026
Hemoglobin (g/dL)	12.9±1.7	10.7±2.3	<0.001
Platelet (10^3^/μL)	315.7±100.2	301.0±107.7	0.606
Neutrophil (10^3^/μL)	11.8±5.3	15.0±6.9	0.007
Lymphocyte (10^3^/μL)	3.0±2.0	9.3±8.7	0.001
Monocyte (10^3^/μL)	1.2±6.5	1.0±0.9	<0.001
Neutrophil-to-lymphocyte ratio	6.2±5.7	2.6±2.5	<0.001
Platelet-to-lymphocyte ratio	145.3±85.0	46.2±25.2	<0.001
Lymphocyte-to-monocyte ratio	4.2±2.9	26.5±56.2	0.177
Mean platelet volume-to-platelet count	0.03±0.02	0.04±0.02	0.421
Blood glucose (mg/dL)	124.7±37.3	289.9±145.6	0.002
ALT (IU/L)	54.5±110.2	273.2±309.7	0.026
AST (IU/L)	100.0±196.5	507.3±577.6	0.026
aPTT (seconds)	28.8±4.4	37.9±12.4	0.020
INR	1.1±0.1	1.5±0.4	0.004
ISS	11.7±7.4	37.0±12.7	<0.001

ALT, alanine aminotransferase; aPTT, activated partial thromboplastin time; AST, aspartate aminotransferase**;** INR, International normalized ratio; ISS, Injury Severity Score.

Logistic regression analysis showed that the NLR (odds ratio [OR], 3.21; *P*=0.048), PLR (OR, 0.90; *P*=0.032), blood glucose (OR, 1.02; *P*=0.024), and ISS (OR, 1.28; *P*=0.011) were independently predictive of mortality risk in PT patients (Table 3).

**Table 3:** Variables Related with Mortality in Logistic Regression Analysis (*n*=358).

Independent Variables	Odds Ratio	95% Confidence Interval	*P* Value
Neutrophil-to-lymphocyte ratio	3.21	1.01–10.22	0.048
Platelet-to-lymphocyte ratio	0.90	0.81–0.99	0.032
Lymphocyte-to-monocyte ratio	2.07	0.97–4.39	0.059
Blood glucose (mg/dL)	1.02	1.00–1.05	0.024
ISS	1.28	1.06–1.54	0.011

ISS, Injury Severity Score.

**Figure 1:**
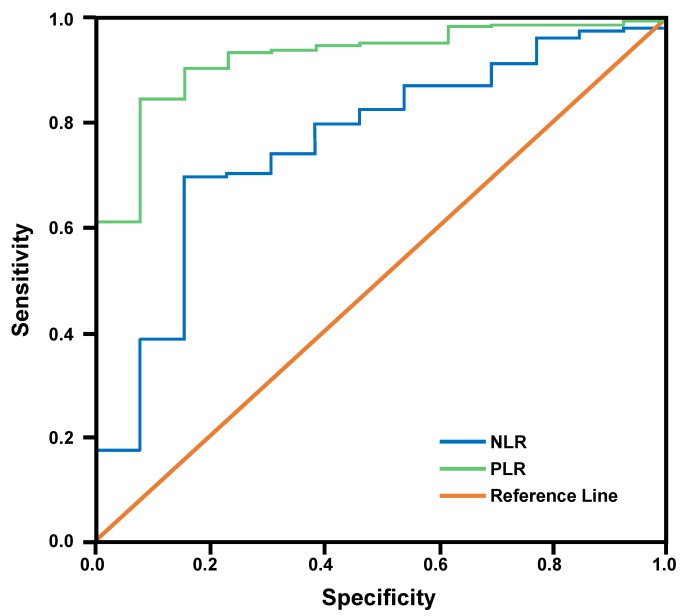
Receiver Operating Characteristic (ROC) Curve of Neutrophil-to-Lymphocyte Ratio (NLR) and Platelet-to-Lymphocyte Ratio (PLR) for the Prediction of Prognosis of Mortality in Patients with Pediatric Trauma.

The ROC curve analysis included the NLR and PLR, which were significantly associated with mortality in bivariate analyses. Referring to Table 4 and Figure 1, the NLR had an area of 0.764, a cut-off of 2.77, a sensitivity of 70%, and a specificity of 77%; the PLR had an area of 0.928, a cut-off of 61.83, a sensitivity of 90%, and a specificity of 85%.

**Table 4:** Cut-off Value, Sensitivity, and Specificity of Neutrophil-to-Lymphocyte Ratio and Platelet-to-Lymphocyte Ratio for Predicting of Mortality in Prognosis of Pediatric Trauma.

Parameter	Neutrophil-to-Lymphocyte Ratio (NLR)	Platelet-to-Lymphocyte Ratio (PLR)
Cut-off value	2.77	61.83
Sensitivity	0.70	0.90
Specificity	0.77	0.85
AUC (95% CI)	0.764 (0.636–0.891)	0.928 (0.871–0.985)

AUC, area under the curve; CI, confidence interval.

## DISCUSSION

This study showed that PT patients with early fatal outcomes had higher blood sugar, and lower PLR and NLR levels on admission, indicating that these parameters may be early predictors of mortality. Moreover, the cut-off values of NLR and PLR have been proposed in this study to estimate the mortality risk in PT patients. Based on a literature review, this is the first study demonstrating a significant association between a high mortality rate and NLR, PLR, and blood glucose among PT patients. As reported in many previous studies, a higher ISS was also predictive of poor outcomes in the present study.^13^,^14^

It should be noted that the cascade of hyper-inflammation of SIRS may occur within 30 minutes after the initial of injury.^15^ Systemic inflammatory response syndrome (SIRS) is one of the most common post-traumatic injury complications and has previously been shown to be a remarkable predictor of mortality in trauma patients.^16^,^17^ Also reported previously, post-traumatic SIRS was not only associated with higher mortality of trauma patients, but also with a higher rate of nosocomial infection and longer length of hospital stay.^17^,^18^ In this regard, it is very important to determine the patients at risk for developing SIRS by identifying reliable biomarkers. In the present study, WBC count, neutrophil count, lymphocyte count, blood glucose, ALT, AST, aPTT, INR, and ISS were remarkably higher in non-survivors, whereas hemoglobin, monocyte count, NLR, and PLR levels were significantly higher in survivors. Logistic regression analysis showed that NLR (OR, 3.21; *P*=0.048), PLR (OR, 0.90; *P*=0.032), blood glucose (OR, 1.02; *P*=0.024), and ISS (OR, 1.28; *P*=0.011) were independently predictive of the risk of mortality in PT patients.

Additionally, this study showed that blood glucose levels were an independent factor for the prognosis of PT patient mortality. This finding was consistent with previous reports indicating that hyperglycemia caused by traumatic brain injury or multiple trauma was associated with poor outcomes in the pediatric population.^19^–^21^

In response to severe tissue trauma such as acute lung injury, neutrophils—as part of a systemic inflammatory response—not only rapidly contribute to inflammation activation, but also may cause organ failure.^22^ Platelets, as cellular effectors of both inflammation and thrombosis, are also activated after traumatic injuries.^23^ As reported in previous studies, the platelet–neutrophil interaction may contribute to neutrophil recruitment, which leads to tissue inflammation.^24^ In addition to contributing to the inflammatory response, a decrease in total lymphocyte count has been found to be related to MODS in adult patients with a traumatic injury.^23^,^25^ However, in the present study, trauma-induced lymphocytosis was observed in the first blood samples taken from PT patients. One possible explanation for this is that a neutrophilic response is expected in some cases of acute and stress-induced inflammatory conditions. However, some stressful and urgent medical conditions, such as non-surgical trauma, cardiac emergencies, sickle cell anemia crises, abdominal pain, and obstetric emergencies, may lead to a transient type of lymphocytosis instead of the neutrophilic response.^26^,^27^

Recently, and similar to this study’s findings, it was reported that, except for patients with post-traumatic SIRS, lymphocytosis and elevated WBC count among patients with traumatic injury were significantly associated with ISS and a higher mortality rate.^28^ Moreover, as demonstrated in some other studies, NLR is closely linked to mortality among patients with severe traumatic injury or critically traumatic brain injury.^29^,^30^

While reduced lymphocyte count is related to immunosuppression, increased platelet and neutrophil counts reflect systemic inflammation. Therefore, NLR and PLR, which combine both immunosuppression and inflammation, might be more reliable biomarkers than WBC or platelet counts.

This study had certain limitations. It was retrospective and included a small number of PT patients; hence the mortality ratio was also relatively low. In addition, laboratory findings for certain clinical and inflammatory markers, such as interleukin-6, TNF-α, etc., were not available. Therefore, the findings of this current study should be validated in further prospective studies. For this purpose, we have a project to verify the results of the study.

## CONCLUSION

In conclusion, the NLR and PLR at the time of admission could be useful predictors for mortality in PT patients. This study indicates that NLR and PLR, as well as blood glucose and ISS at the time of admission, were independently associated with post-traumatic SIRS. Further studies should be carried out to validate the role of those parameters to optimize treatment strategy in PT patients.

## Abbreviations

ALT: alanine aminotransferase
aPTT: activated partial thromboplastin time
AST: aspartate aminotransferase
AUCs: areas under the curve
ED: emergency department
Hb: hemoglobin
hcDNA: histone-complexed DNA
INR: international normalized ratio
ISS: Injury Severity Score
MODS: multi-organ dysfunction syndrome
NLR: neutrophil-to-lymphocyte ratio
PLR: platelet-to-lymphocyte ratio
PT: pediatric trauma
ROC: receiver operating characteristic
SIRS: systemic inflammatory response syndrome
WBC: white blood cell.
